# Mechanical Behaviors of Flax Fiber-Reinforced Composites at Different Strain Rates and Rate-Dependent Constitutive Model

**DOI:** 10.3390/ma12060854

**Published:** 2019-03-13

**Authors:** Dayong Hu, Linwei Dang, Chong Zhang, Zhiqiang Zhang

**Affiliations:** 1 Department of Aircraft Airworthiness Engineering, School of Transportation Science and Engineering, Beihang University, Beijing 100191, China; hudayong@buaa.edu.cn (D.H.); danglinwei@buaa.edu.cn (L.D.); chongzhang@buaa.edu.cn (C.Z.); 2 Aircraft/Engine Integrated System Safety Beijing Key Laboratory, Beijing 100191, China; 3 Beijing Key Laboratory of Rehabilitation Technical Aids for Old-Age Disability, Key Laboratory of Rehabilitation Technical Aids Analysis and Identification of the Ministry of Civil Affairs, National Research Center for Rehabilitation Technical Aids, Beijing 100176, China; 4 Qinhuangdao Institute of National Research Center for Rehabilitation Technical Aids, Qinhuangdao 066000, China

**Keywords:** flax fiber-reinforced composite, strain rate effect, Johnson–Cook model, lattice structure, failure mechanism

## Abstract

Flax fiber-reinforced composites (FFRCs) exhibit excellent environmentally friendly qualities, such as light weight, low cost, recyclability, and excellent mechanical properties. Understanding the dynamic mechanical behavior of FFRCs could broaden their potential applications in lightweight, crashworthy, and impact-critical structures. This study presents a study on the fabrication of FFRCs by vacuum-assisted resin infusion. The dynamic stress–strain responses of the fabricated specimens at strain rates ranging from 0.006 $s^{-1}$ to 2200 $s^{-1}$ were evaluated using quasi-static tests and the Split–Hopkinson pressure bar (SHPB). The results indicated that the FFRC exhibited superior strain rate sensitivity. Final deformation photographs and scanning electron micrographs clearly revealed the damage evolution of the FFRC specimens, as well as various failure mechanisms, including fiber–matrix debonding, fiber pull-out, and fiber fracture at different strain rates. On the basis of the experimental results, a simplified Johnson–Cook model was established to describe the strain-rate dependent constitutive model of FFRC. The validation of the suggested constitutive model was embedded in the finite element simulations and could well repeat the strain wave observed from the experiment results. Finally, the quasi-static compression and drop-hammer impact of pyramidal lattice structures with FFRC cores were investigated both numerically and experimentally, proving the effectiveness of the simplified Johnson–Cook model. This study could potentially contribute to a deeper understanding of the dynamic mechanical behavior of FFRCs and provide fundamental experimental data for future engineering applications.

## 1. Introduction

Over the last decades, lightweight, low cost, and recyclability have been the highlighted material properties for automotive and aerospace industries [1]. Synthetic high-performance fiber-reinforced composites were able to overcome the lightweight problem; however, most of them were nondegradable and harmful to the environment. To reduce the environmental impact of composite materials, natural fiber-reinforced composites (NFRCs) with favorable mechanical and economic properties, as well as higher environmental friendliness, have been investigated as potential substitutes for synthetic fibers as reinforcements [1,2,3,4,5,6,7,8,9]. Extensive research on the mechanical properties of NFRCs have been conducted and compared with those of synthetic fibers. Dittenber and Gangarao [1] reported that the specific modulus of flax fiber was approximately 45$\;\mathrm{GPa}/\left(g/{\mathrm{cm}}^{3}\right)$, higher than that of glass fiber measuring approximately 29 $\mathrm{GPa}/\left(g/{\mathrm{cm}}^{3}\right)$. Joshi et al. [10] showed that flax fibers exhibited specific strength equal to or even higher than that of glass fibers and could be a suitable replacement for glass fibers. Amenini et al. [6] investigated the dynamic characterization of a flax fiber-reinforced polyamide 11 bio-composite. Koh and Madsen [11] studied the failure criteria to accurately predict the strength of NFRCs, and they recommended Hashin and Puck failure theories, as they showed the smallest error compared to experimental data. Ramesh [12] carried out a detailed review on the preparation and properties of flax fiber and its composite materials.

In recent years, NFRCs have been investigated for application in aeronautic and automotive engineering [4,13,14] because of their distinct mechanical properties and low cost. Composites in these fields have been highly susceptible to impact damage induced by striking with foreign objects [13,15,16]. Thus, characterizing the mechanical performance of NFRCs under impact loading conditions is important. Ravandi et al. [17] reported that composites with woven flax fiber exhibited better impact properties compared to those with non-woven fiber. Meredith et al. [18] performed drop hammer experiments at an impact speed of approximately 8.0 m/s to evaluate the energy absorption characteristics of cone tubes made of woven flax and regenerated cellulose textiles; the specific energy absorption of cone tubes varied from 21.2 to 34.2 kJ/kg. Another important finding was that cone tubes made of Biotex flax combined with stiffer resin could obtain a higher specific energy absorption than that of cone tubes made of tougher resin. López–Alba et al. [4] investigated the energy absorption behaviors of NFRC tubes at different impact velocities. They found that the specific energy absorption of NFRC tubes heavily depended on the material parameters, including wall thickness, type of weave material, reinforced textiles, and matrix used. Shishevan et al. [19] assessed the low-velocity impact responses of basalt fiber-reinforced composites at different magnitudes of impact energy—30, 60, 80, 100, 120 and 160 J. In addition, the related key impact parameters, such as maximum contact force, absorbed energy, deflection, and duration were compared with those of carbon fiber-reinforced composites. On the basis of the experimental results, the impact performance of basalt fiber-reinforced composites was greater than that of carbon fiber-reinforced composites because of the higher toughness of basalt fibers. To evaluate the effects of fiber content and fiber orientation on the impact behavior of flax fiber-reinforced polypropylene composites, Rahman et al. [20] measured the impact properties of FFRCs by using the Charpy impact test and drop-weight impact test. Their results indicated that composites with varying fiber orientations exhibited different energy absorption for the in-plane and out-of-plane impact loads. Dhakal et al. [21] evaluated the effects of impactor shape and impact velocity on the dynamic mechanical properties of hemp–unsaturated PE composite under low-velocity impact conditions. The results showed that the specimens impacted by a hemispherical impactor exhibited higher force and absorbed more energy compared with specimens impacted by a conic impactor. In addition, with an increase in impact velocity, the damage to the back face of the specimen was more prominent for the laminates impacted by the hemispherical impactor. Rajaei et al. [22] investigated the effect of preheating on the impact performance of flax fiber-reinforced composite laminates. The impact test results showed that heat exposure at 300 °C reduced the energy absorption of the flax fiber composites. Shen et al. [23] also studied the effect of manufacturing process temperature, exposure temperature and water absorption on the low-velocity impact damage threshold and damage mechanisms of NFRCS. It was found that excessive temperature and water uptake could cause a serious reduction on the impact damage threshold and damage resistance. To further enhance impact properties of flax fibers, Al-Hajaj et al. [24] carried out pendulum impact tests with a range of impact energies (5–40 J) to investigate the effect of hybridization with woven carbon fibers plus flax fibers on impact properties. Results showed that these hybrid composites had superior impact properties compared to pure flax fiber-reinforced epoxy composites, suggesting that hybridization using synthetic and natural fiber could be done successfully. Moreover, under high loading rate conditions, the effect of strain rate on the mechanical properties of NFRCs has received considerable interest and has been extensively studied in metal, foam, and carbon- and glass fiber-reinforced composites [25,26]. Using the Split–Hopkinson pressure bar (SHPB), Omar et al. [27] examined the dynamic properties of pultruded jute and kenaf fiber-reinforced unsaturated polyester composites under different strain rates, nearly reaching 1400 $s^{-1}$. The compression modulus, compressive strength, and flow stress of both NFRCs were highly sensitive to strain rates. Similar experiments were also performed by Kim et al. [28] to study the dynamic mechanical responses of hemp, hemp/glass hybrid, cellulose, and wheat straw-reinforced polymeric composites at strain rates between 600 and 2400 $s^{-1}$. These NFRCs exhibited evident strain rate sensitivity. However, studies concerning the effects of strain rate on the mechanical behaviors of NFRCs compared with those of metals and composites, remained relatively inadequate, requiring further studies. 

In addition, numerical simulation has become a widely used tool for predicting structural responses, thereby reducing experiment expense and time cost. For NFRCs, several numerical modeling studies can be found in the literature [29,30,31,32]. On the basis of the hypotheses of linear–elastic behavior up to failure and strain rate independent behavior, Rubio-López et al. [29] developed a finite element method (FEM) model to predict the low-velocity impact behavior of all-cellulose composite plates. Poilâne et al. [30] proposed a viscoelastoplastic model with eight independent parameters to model the behavior of the unidirectional flax fabric polymer composite. However, these two models failed to consider strain dependency. Rubio-López [31] presented a rheological model to describe the viscoplastic behavior of NFRCs at different strain rates and used this model to successfully analyze the low-velocity impact responses of NFRCs. Numerical prediction was consistent with experimental data conducted with two impactor shapes at different impact energies. However, in their study, the strain rates were considerably low, ranging from ${2. 08\;\times \;10}^{-4}{\;s}^{-1}$ to ${8.33\;\times \;10}^{-3}{\;s}^{-1}$, which could still be considered under quasi-static loading condition. Consequently, higher strain rates were expected to more accurately describe the dynamic behaviors of NFRCs.

To bridge these gaps, in the present study, flax fiber-reinforced composites (FFRCs) were manufactured by vacuum-assisted resin infusion (VARI). Quasi-static and SHPB experiments were then conducted to obtain mechanical properties in order to investigate the effects of strain rate. Based on experimental results, a simplified Johnson–Cook model, validated by SHPB experiments, was developed to describe both quasi-static and dynamic characteristics of FFRCs. Lastly, the effectiveness of the simplified Johnson–Cook model was further verified by quasi-static and drop-hammer crushing experiments of lattice structures.

## 2. Materials and Methods 

### 2.1. Fabrication

Flax fiber plain weave fabrics (50% weft yarns per 50% warp yarns: $\left[0{}^{\circ}/90{}^{\circ}\right]$ purchased from Linyi City, Shandong Province, China) and epoxy resin (LY1564/Aradur22962, Huntsman, from Shanghai, China) were selected to fabricate laminated composites by VARI. The diameters of the tows of the fabrics varied from about 0.6 mm to 1.5 mm. The nominal area density of the fabric and the fabric weight fraction were measured to be approximately 230 $g/m^{2}$ and 48%, respectively. The quasi-static tension tests of the pure matrix were performed and the stress-strain curves were provided in Figure S1. The viscosity of the resin was 450 mPa·s and the glass transition temperature was 140°C. The operating time of the resin was about 120 min, within which the fabrication should be finished outside the heating oven. The curing cycle was 80 °C for 2 h and 120 °C for 3 h.

**Figure 1:**
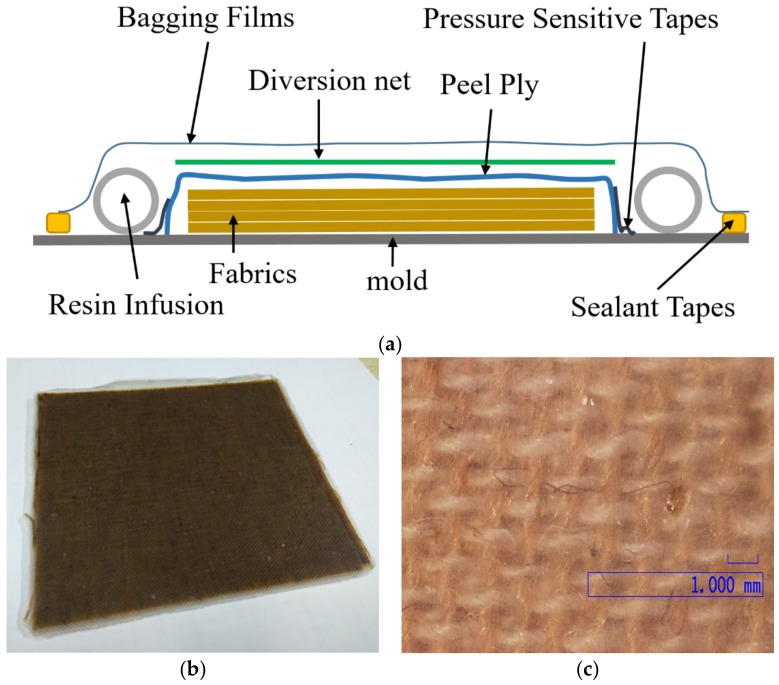
(**a**) Schematic of woven fiber and other supplementary materials; (**b**) composite sheet; (**c**) detailed image of the sheet.

VARI is a type of low-cost molding technology for manufacturing large-scale composite structures. The process in this study includes the following four steps:

◇ First, the mold surface is cleaned with acetone to achieve a perfect surface quality.
◇ Secondly, the layers of woven fiber dried at 70 °C for 3 h in an oven are laid on a mold sheet and other supplementary materials as shown in Figure 1a.
◇ Third, the resin is injected into the mold with atmospheric pressure after vacuuming and checking the sealing.
✧ Last, the resin flow is closed, and the resin is cured in an oven. The composite sheet after demolding is shown in Figure 1b, and the detailed image of the sheet is shown in Figure 1c.

**Figure 2:**
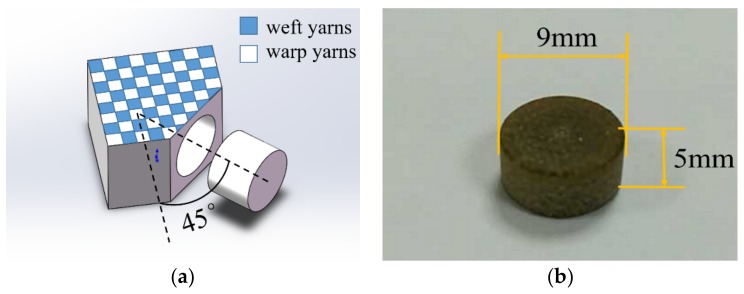
Cutting direction (**a**) and size (**b**) of the material test specimen.

In the present study, the fabricated composite sheet consisted of 20 layers, with a total thickness of 11.0 mm. The measured density of the composite sheet was 1.18$\;g/{\mathrm{cm}}^{3}$. Cylindrical specimens with measuring 9 mm in diameter and 5 mm in length were adopted for both quasi-static and dynamic compression experiments. For consistency with the fiber orientation of the trusses of the lattice structure in Section 4.3, the same direction with a fiber orientation of 45° was used for the specimens of quasi-static and SHPB compression experiments (Figure 2). 

### 2.2. Experimental Set-Up

#### 2.2.1 Quasi-Static Compression Experiment

Quasi-static compression experiments were conducted on a hydraulic servo testing machine INSTRON 8801 (manufactured in Boston, MA, USA) at constant loading rates of 2, 20, and 200 mm/min, corresponding to the nominal strain rates of 0.006, 0.06, and 0.6 $s^{-1}$, respectively. The temperature was 25 °C, the relative humidity was 20%, and the maximum load capacity of the machine was 100 kN. The specimens were placed centrally between the polished platens. Prior to continuous loading, about 10 N was preloaded to eliminate the clearance. The axial deformation was captured by a non-contacting video extensometer (Instron AVE 2.0, manufactured in Boston, MA, USA) with a precision of 0.5 per thousand during the quasi-static tests.

#### 2.2.2. SHPB Experiment

**Figure 3:**
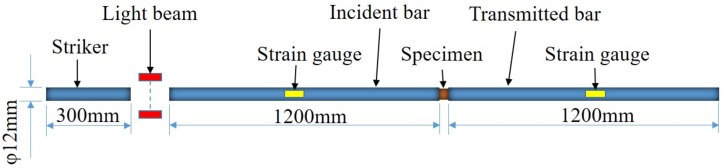
Schematic of the SHPB device.

The SHPB apparatus is widely used to investigate the dynamic behavior of materials. The device typically consists of a striker bar, an incident bar, a transmission bar, and a gas gun, among other things, as shown in Figure 3. The strain gauge, digital storage oscilloscope, and ultrahigh dynamic extensometer are used to calibrate and measure the time history curves of incident, reflected, and transmitted waves. The cylindrical specimen is placed between the incident bar and the transmission bar. When the striker is propelled from the gas gun to impact the incident bar, a compressive elastic wave is generated and propagated through the incident bar. Once the wave reaches the specimen, part of the wave reflects on the interface of the specimen, while the remaining portion passes through the transmission bar. The traveling waves in the incident bar and the transmitted bar can be quantitatively captured by strain gauges mounted on these two bars. Thus, the strain–time histories of the incident, reflected, and transmitted waves can be recorded using the oscilloscope.

The engineering stress, engineering strain, and strain rate can be calculated using the following equations [23]:

(1a) $$\sigma_{e}\left(t\right)=\frac{EA_{0}}{A_{s}}\varepsilon_{t}\left(t\right),$$

(1b) $$\varepsilon_{e}\left(t\right)=-\frac{2C_{0}}{L_{s}}\int_{0}^{t}\varepsilon_{t}\left(t\right)dt,$$

(1c) $$\dot{\varepsilon }\left(t\right)=-\frac{2C_{0}}{L_{s}}\varepsilon_{r}\left(t\right),$$

where E, $C_{0}$, and $A_{0}$ denote Young’s modulus, stress wave speed, and the cross-section area of the incident bar, respectively. $A_{s}$ and $L_{s}$ represent the cross-section area and length of the specimen, respectively. $\varepsilon_{t}\left(t\right)$ and $\varepsilon_{r}\left(t\right)$ refer to the amplitude of the transmitted wave and the reflected wave as functions of time *t*, respectively. $\sigma_{e}\left(t\right)$ and $\varepsilon_{e}\left(t\right)$ denote engineering stress and strain, respectively. 

Furthermore, the true stress–strain relationship can be obtained using the following equations:

(1d) $$\sigma \left(t\right)=\sigma_{e}\left(t\right)\left(1-\varepsilon_{e}\left(t\right)\right),$$

(1e) $$\varepsilon \left(t\right)=\ln\left(1-\varepsilon_{e}\left(t\right)\right),$$

where $\sigma \left(t\right)$ and $\varepsilon \left(t\right)$ are the true stress and the true strain, respectively. 

In the present study, the detailed parameters of the SHPB apparatus were as follows: the striker measured 300 mm in length and 12 mm in diameter; both the incident bar and the transmitted bar were 1200 mm in length and 12 mm in diameter; and the three bars were made of steel. The experiments were conducted at different strain rates: 1300${\;s}^{-1}$ and 2200${\;s}^{-1}$. Two repeated experiments were conducted at each strain rate to ensure the repeatability of the experimental results.

## 3. Experimental Results

**Figure 4:**
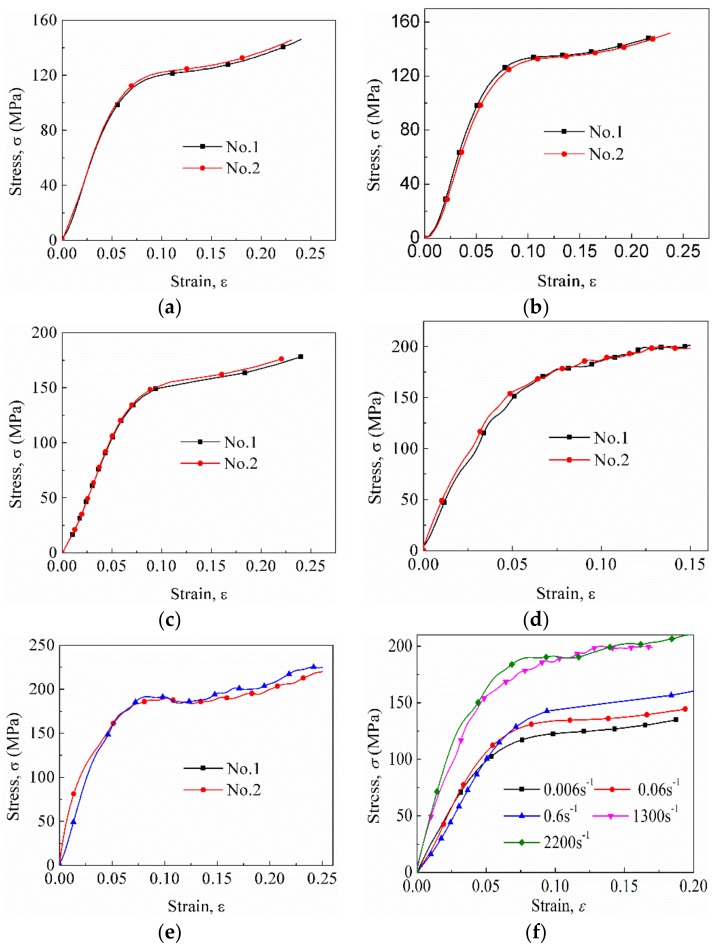
Stress–strain curves at different strain rates: (**a**) 0.006${\;s}^{-1}$, (**b**) 0.06${\;s}^{-1}$, (**c**) 0.6${\;s}^{-1}$, (**d**) 1300${\;s}^{-1}$, (**e**) 2200${\;s}^{-1}$. (**f**) Comparison of typical curves.

In the SHPB tests, the striker bar was launched by varying the gas pressure to achieve different average nominal strain rates. Figure 4 shows the stress–strain curves of the FFRCs under quasi-static and SHPB compression experiments. The experimental results exhibited good repeatability (see Figure 4a–e). In addition, Figure 4f shows that FFRC is strongly affected by strain rates. The yield stress, as well as the flow stress, of the FFRCs markedly increased with an increase in strain rate. Therefore, the FFRCs showed evident strain rate sensitivity. This finding was highly similar to that reported by Omar et al. [27].

**Figure 5:**
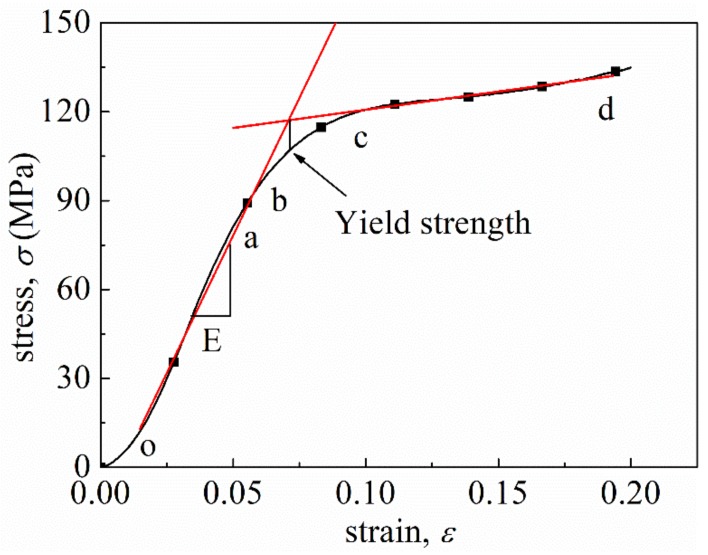
Different stages of a typical stress–strain curve: elastic region (oa), yield stage (bc), and plastic stage (cd).

The representative stress–strain curve in Figure 5 shows the trend of this stress–strain curve can be divided into three distinct stages—elastic region (oa), yield stage (bc), and plastic stage (cd)—similar to that of metal [25]; however, the specimen is made of a fiber-reinforced polymer material. The yield strength, $\sigma_{1}$, of the representative curves were extracted from the figure and listed in Table 1, consistent with the previous study (100–200 MPa) [33]. Evidently, $\sigma_{1}$ increased with an increase in strain rate. For example, when the nominal strain rate reached 1300 $s^{-1}$, $\sigma_{1}$ markedly increased to 152 MPa, which was about 1.5 times higher than that of 0.006 $s^{-1}$. The increase in yield strength from 1300 $s^{-1}$ to 2200 $s^{-1}$ was 11.7 MPa.

In addition, Young’s modulus exhibited an appreciably increasing trend with an increase in strain rate, which was consistent with reference [27]. They attributed the increase in stiffening to the increase in strain rate, thereby decreasing the molecular mobility of polymer chains. However, dynamic Young’s modulus could not be accurately measured by SHPB [34]. In the current study, the value of Young’s modulus was not given to avoid inconsistency.

**Figure 6:**
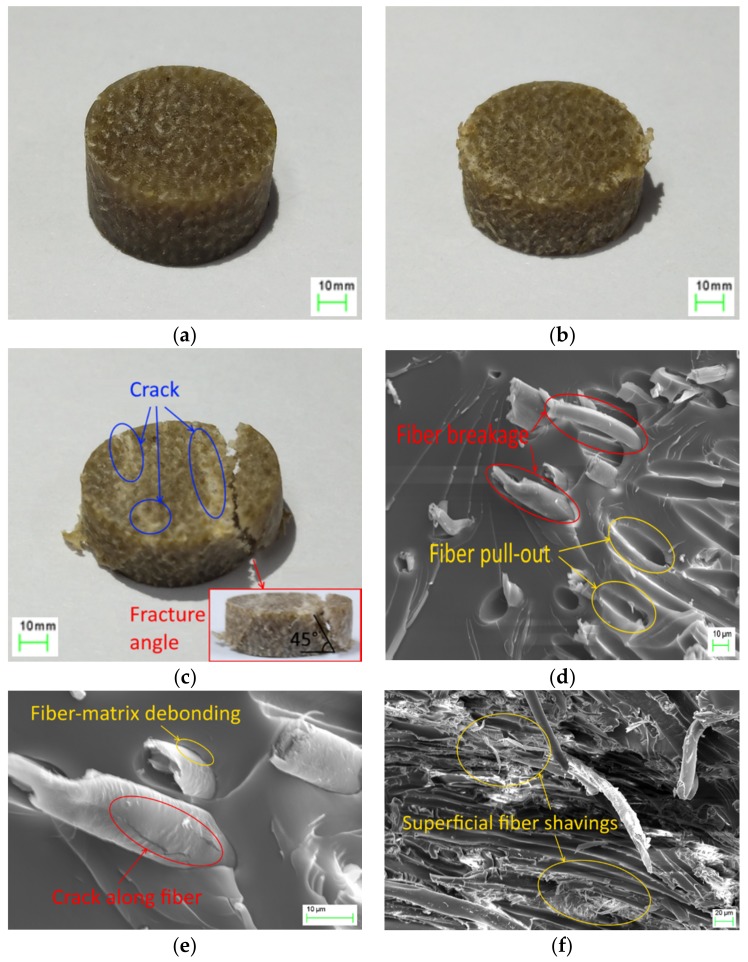
Specimens after experiments (**a**) 0.6 $s^{-1}$, (**b**) 1300 $s^{-1}$, (**c**) 2200 $s^{-1}$; Micrographs of fractured edges: (**d**) fiber breakage and fiber pull-out, (**e**) fiber–matrix debonding and crack along the fiber, (**f**) superficial fiber shavings.

Figure 6a–c shows the final deformation morphologies of the crushed specimens. At a lower strain rate, the specimen only exhibits a reduction in thickness, where no obvious damage in appearance is observed. In the case of 1300${\;s}^{-1}$, the margin of the specimen was damaged to a certain extent and compression deformation was evident. In the case of 2200${\;s}^{-1}$, the specimen broke into two fragments, along with small cracks, and the fracture angle was approximately 45° (Figure 6c). The magnitude of the shear fracture angle mainly depended on the interfacial bond strength: a strong interface resulted in a larger shear fracture angle, whereas a weak interface generated a small fracture angle [27]. The failure could be inferred to have been initiated by matrix plasticity, followed by cracks passing through the layers of the laminate and forming a shear fracture with an angle of 45°. 

The microscopic failure mechanism was analyzed by scanning electron microscopy (SEM, Zeiss Auriga, manufactured in Oberkochen, Germany) to highlight the dominant failure modes at selected locations on the specimens. Prior to SEM observation, the specimens were coated with an ion sputter coater to obtain enhanced conductance. Figure 6d–f presents micrographs of the fractured surface of the crushed specimen at the strain rate of 2200 $s^{-1}$. Fiber pull-out from the matrix is clearly shown in Figure 6d. Almost no matrix residue could be found on the surface of the fibers (Figure 6d,e). This observation could be attributed to the poor adhesion between the hydrophilic flax fiber and the hydrophobic epoxy matrix [18,35]. As seen in Figure 6e, a crack occurs along the fiber’s longitudinal direction, which could be attributed to the shear failure of the fiber when the matrix fractured. The crack also indicated a reduction in the shear strength of the flax fiber. In Figure 6f, flax fragments were stuck to the matrix after fiber pull-out, and superficial flax shavings exhibit partly separated from the fibers—that is, not completely from the reinforcements. This occurrence was highly consistent with the observation of Liang et al. [35] that this could be considered as an additional type of damage mechanism for NFRCs. 

## 4. Discussion

### 4.1. Simplified Johnson–Cook Model

The simplified Johnson–Cook model is widely accepted to describe the coupling factors among stress, strain, and strain rate [25,36]. The profile of the stress–strain curves of FFRCs was similar to those of traditional metals with a well-defined Johnson–Cook model. Owing to the difficulty in extracting temperature data and the slight effect of temperature on constitutive behaviors under low impact energy, for the sake of simplicity, only isotropic hardening and strain-rate hardening effects were considered in this study. Therefore, the dynamic behavior of FFRCs can be expressed as

(2) $$\sigma =\left(A+B\varepsilon^{n}\right)\left(1+C\ln{\dot{\varepsilon }}^{*}\right),$$

where σ is the stress; A is the yield stress; B and n represent the effect of strain hardening, respectively; C is the material constant determined by the specific material, representing the strain rate dependence of the material; ε is the equivalent plastic strain and obtained by subtracting the elastic strain from the total strain; $\dot{\varepsilon }$ is the strain rate; and ${\dot{\varepsilon }}^{*}$ is the dimensionless plastic strain rate expressed as $\dot{\varepsilon }/{\dot{\varepsilon }}_{0}$, where ${\dot{\varepsilon }}_{0}=$ 0.006 $s^{-1}$ on the basis of quasi-static experiments. 

Naturally, in the quasi-static experiment for ${\dot{\varepsilon }}^{*}=1$, the constitutive model of Equation (2) can be further simplified to

(3) $$\sigma =A+B\varepsilon^{n},$$

Taking the logarithm of both sides of Equation (3) may result in the following:

(4) $$\ln\left(\sigma -A\right)=\ln B+n\ln\varepsilon ,$$

Subsequently, Equation (4) is applied to fit the quasi-static experimental data in logarithmic coordinates by the least square method, such that lnB represents the intercept of the straight line, and n represents the slope. Thus, B and n can be determined using simple mathematical conversion. At room temperature, C can be obtained through the fitting in accordance with Equation (2)

(5) $$\frac{\sigma_{2}\left(\dot{\varepsilon }\right)}{\sigma_{1}}-1=C\ln\frac{\dot{\varepsilon }}{{\dot{\varepsilon }}_{0}},$$

where $\sigma_{1}=A+B\varepsilon^{n}$, $\sigma_{1}$ represents the yield stress when the strain rate is $0.006{\;s}^{-1}$, and $\sigma_{2}\left(\dot{\varepsilon }\right)$ is the yield stress at the strain rate of $\dot{\varepsilon }$.

**Figure 7:**
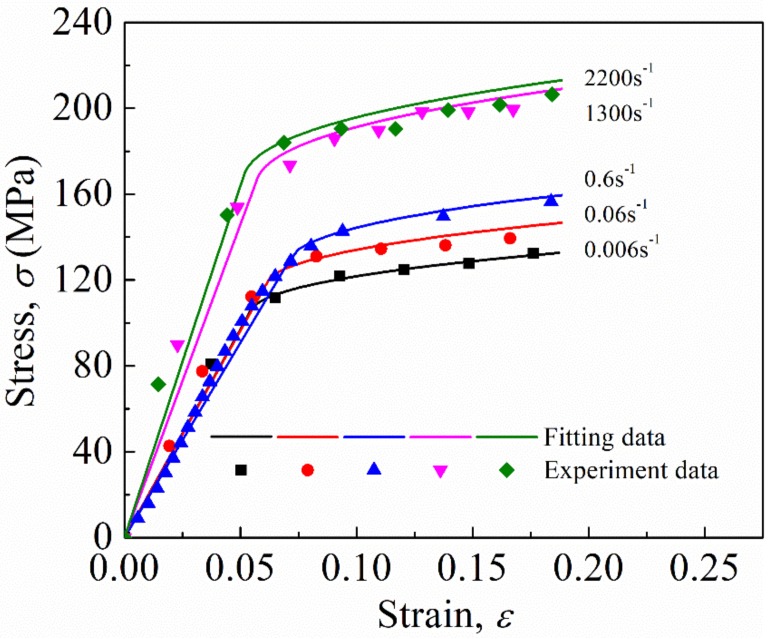
Comparison of the fitting curves of the experimental data.

The simplified Johnson–Cook model (Equation (2)) was used to describe the dynamic rate-dependent constitutive behavior of the FFRCs. The fitting parameters in the constitutive models in accordance with the experimental data are listed in Table 2 and the constitutive relationship was obtained as follow: $\sigma =\left(102.0+70.8\varepsilon^{0.416}\right)\left(1+0.047\ln\frac{\dot{\varepsilon }}{0.006}\right)$. The fitting curves of the model and the experimental data are illustrated and compared in Figure 7.

### 4.2. Dynamic Wave Verification

To validate the effectiveness of the developed simplified Johnson–Cook model of FFRCs, numerical simulation was conducted using the commercial FEM software ABAQUS 6.13 (Dassault Systemes S.A, Vélizy-Villacoublay, France) to simulate the SHPB experiment. 

A three-dimensional FEM model was set up to simulate the SHPB experiments. The FEM model consisted of four components: a striker bar, an incident bar, a transmission bar, and an FFRC specimen, each of which was of the same size as the SHPB apparatus shown in Figure 3. To improve the accuracy of the FEM analysis, an 8-node linear brick with reduced integration and hourglass control (C3D8R) was adopted. The minimum size of all elements was 1 mm. The same material was used for the three components made of steel, with the following measurements: modulus, 190 Gpa; density, 8 $g/{\mathrm{cm}}^{3}$, and Poisson ratio, 0.3. The developed simplified Johnson–Cook model was used to simulate the mechanical behavior of the FFRC specimen in Table 2. The time history of the strain wave was obtained from the same location of the strain gauges on the incident and transmission bars with SHPB experiments. 

**Figure 8:**
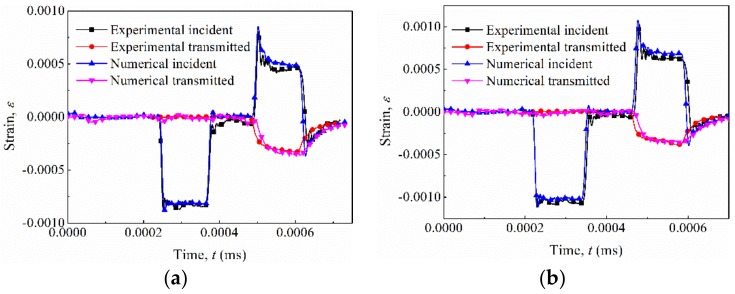
Comparison of strain waves between the simplified Johnson–Cook model and experimental data under strain rates (**a**) 1300 $s^{-1}$, (**b**) 2200 $s^{-1}$.

The speed of the striker was varied to obtain the various loading conditions of the tested specimens. The strain wave obtained from the experiments, with its counterpart from the FEM calculations, is shown in Figure 8. Consistency was found, indicating the validity and accuracy of the simplified Johnson–Cook material model. However, a slight general difference still observed, which could be attributed to the following: (a) The influence of thermal softening under impact could not be simulated in ABAQUS Explicit [25]; (2) The geometry of the specimens was not perfectly cubic [37]; (3) The non-parallelism and friction between the faces in contact with the bars were hardly included in the FEM model [37]; (4) The strain rate was not constant in the SHPB, and so on. 

### 4.3. Prediction of the Crushing Peofrmance of Lattice Structures

As in Section 4.2, to further validate the effectiveness of the developed simplified Johnson–Cook model of the FFRC applied in engineering structure analysis, numerical simulation was also conducted using ABAQUS to simulate lattice structures subjected to quasi-static crushing and drop-hammer impact.

#### 4.3.1. Specimen and FEM Model

**Figure 9:**
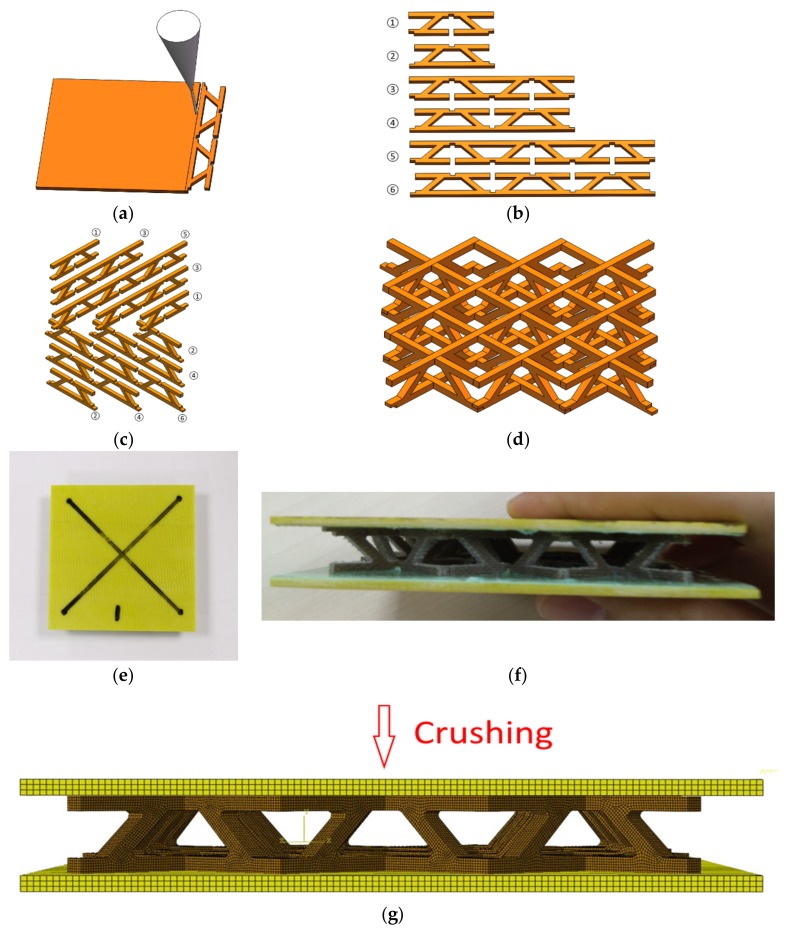
Pyramid lattice manufacturing: (**a**) Cutting truss strips of the lattice core from the fabricated FFRC; (**b**) obtaining six types of truss; (**c**) assembling truss into a pyramidal lattice structure by strip slot insertion; (**d**) assembled lattice structure; (**e**) top view of the specimen; (**f**) left view of the specimen; (**g**) FEM model of the specimen.

Lattice structures were man-made open, porous cellular solids with periodic truss microstructures [38,39], which could meet many stringent requirements of engineering applications, such as blast and ballistic resistance, impact load carrying, and energy absorption. In the present study, FFRCs were used to manufacture pyramidal lattice cores with additional horizontal trusses for structure crashworthiness applications. The manufacturing process is illustrated in Figure 9. Six types of truss strips were cut with a carving machine (3040, Shenzhen Yidiao, Shenzhen, China) with a cutting precision of 0.03 mm from an FFRC sheet and then assembled into a pyramidal lattice structure by strip slot insertion (see Figure 9a–d). To fix the lattice core, two pieces of glass fiber-reinforced composite panels (see Figure 9e) were used to bond both sides of the lattice core forming a sandwich structure (see Figure 9f). The specimen of the lattice structure measured 84 mm in length, 84 mm in width, and 14 mm in height. 

Numerical models were then established to predict the crushing performance of the lattice structure under quasi-static and drop-hammer impact conditions in the out-of-plane loading direction. A three-dimensional FEM model with the C3D8R element type was numerically established in ABAQUS, (Figure 9g). The density of the elements was relatively higher in the core, and the element size was 0.3 mm. The FEM model had exactly the same size as the tested specimen with setups. Two plates were considered as rigid bodies to crush the specimen. 

#### 4.3.2. Quasi-Static and Impact Experiments

Quasi-static and drop-hammer impact experiments, corresponding to the loading condition the of numerical simulation, were also conducted to validate numerical results to further evaluate the effectiveness of the simplified Johnson–Cook model in typical engineering structures. Quasi-static crushing tests were conducted on INSTRON 8801 (manufactured in Boston, MA, USA), where specimens were placed centrally between the polished moving platen and the stationary platen. To eliminate the influence of glass fiber panels, the platens were bigger than the specimens. All specimens were crushed under the following conditions: stroke distance, 5 mm; loading rate, 2 mm/min; and temperature, 25 °C. The crush-load curve with respect to the moving platen displacement was recorded automatically into a computer. A drop-hammer testing system was used for impact crushing tests to evaluate the effect of strain rate. The drop hammer, with a mass of 6 kg, was lifted by the pulley to different heights and then released through the trip gear to achieve different levels of impact energy, such as 30 and 45 J. The strain rates during impact crushing fell within the range of the SHPB experiments. The specimens were located at the center of the base of the drop-hammer testing system, directly opposite to the center of the mass of the hammer. The size of the hammer was larger than that of the specimen. A force sensor was mounted on the base to measure the impact force. The force response signals could then be recorded using a digital oscilloscope at a sampling frequency of 100,000 Hz and stored on a computer. Thus, the time history of crush load could be obtained from the recorded data. In addition, during the impact tests, a high-speed camera was used to capture the crushing deformation of the specimen with a rate of 2000 frame/s.

#### 4.3.3. Numerical Simulation and Experiment Correlation

**Figure 10:**
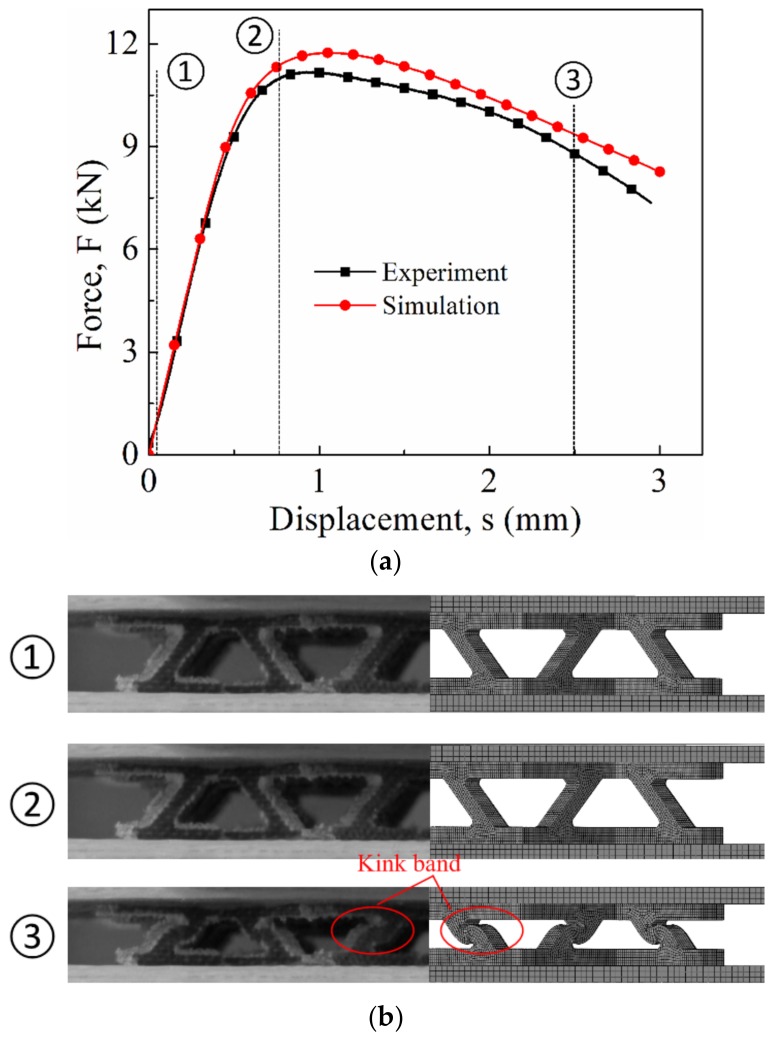
Comparison between the experiment and the numerical simulation: (**a**) Crush load versus displacement curve; (**b**) deformation process.

**Figure 11:**
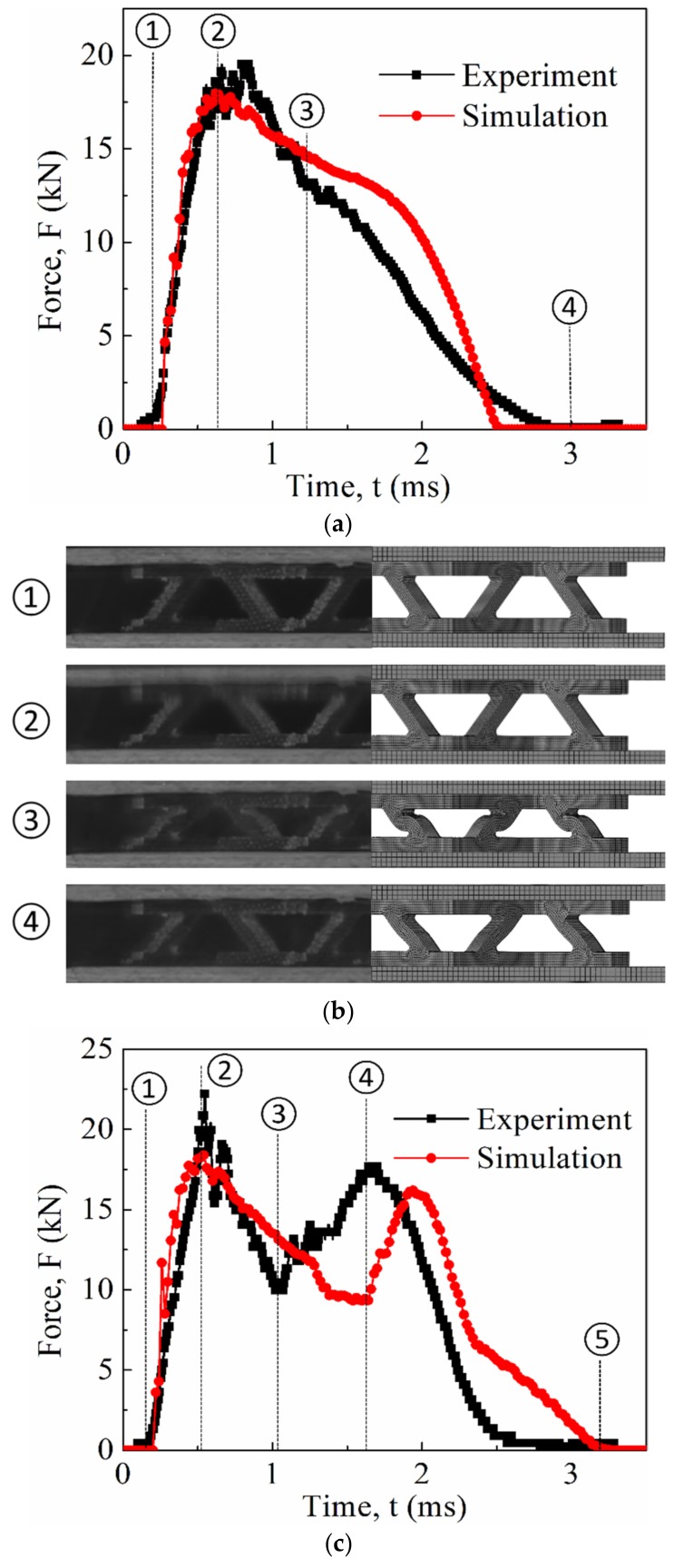

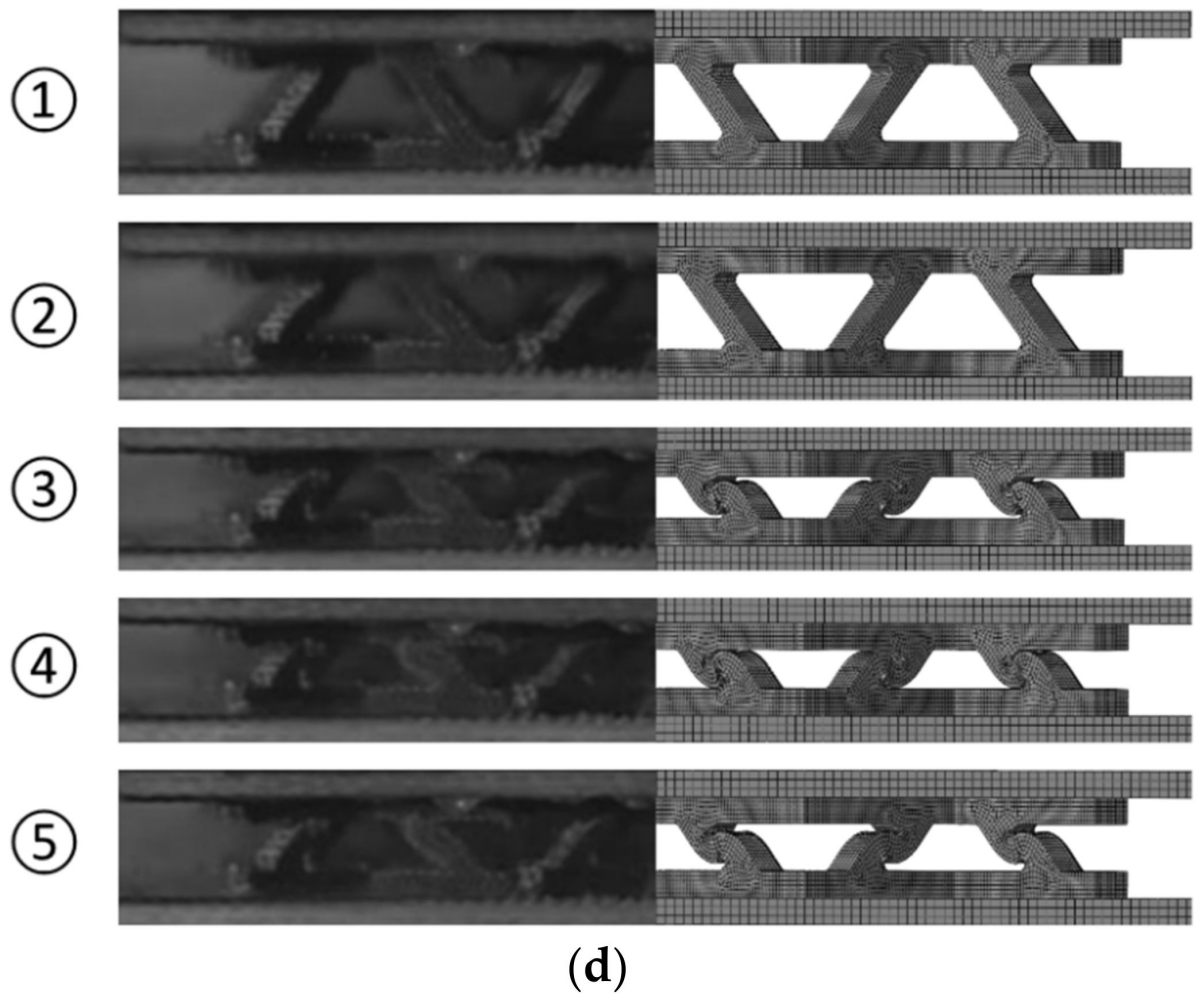
Comparison of the time history of crush load and deformation obtained by FEM calculation with its counterpart from experimental measurement under different impact energy: 30 J (**a**,**b**) and 45 J (**c**,**d**).

Numerical simulation of lattice structures under quasi-static and drop-hammer impact conditions, as well as the corresponding experiments, was conducted. The results are depicted in Figure 10 and Figure 11. At a glance, an acceptable agreement could be observed for both quasi-static and dynamic results, indicating the validity of the simplified Johnson–Cook model. Table 3 summarizes the peak load comparison between the numerical simulation and the experiments. 

Under quasi-static conditions, the specimen exhibited typical progressive crushing (Figure 10). When the moving platen came into contact with the specimen, the crushing load was generated and increased linearly until the peak load was reached, corresponding to the critical Euler buckling load of the truss. As compression continued, the crush load was followed by a decrease stage and kink-band formation, resulting in the development of shear stresses, as featured in Figure 10b. In Figure 10a, the crush load curves of both numerical and experimental results exhibited a highly similar trend, except that the former was slightly higher than the latter. The deformation process was also similar with each other; specifically, numerical simulation was able to well predict the kink band formation of the specimen.

In the drop-hammer impact experiments, dynamic crushing was highly similar to the quasi-static crushing with the same failure mechanism of Euler buckling. Figure 11 presents the time history of crush load of both the numerical and experimental results under impact energy of 30 and 45 J, and corresponding deformation results were also presented. The numerical results were close to the experimental results, with a slight difference. In addition, the peak load was significantly higher than the quasi-static crushing experiment, which illustrates the effect of strain rate. As for the lattice structure subjected to crush loading with impact energy of 30 J, the curves of both the numerical simulation and the experiment were clearly characterized by one peak corresponding to Euler buckling initiation (Figure 11a). The deformation of the numerical simulation was also consistent with that of experiments (Figure 11b). With regard to the lattice structure subjected to crush loading with an impact energy of 45 J, both curves of the numerical simulation and the experiment were clearly characterized by two peaks (Figure 11c). The first peak load also corresponded to Euler buckling initiation. The second peak load was attributed to the impact between the top and bottom panels, when higher impact energy beyond the truss bearing capacity resulted in lattice compaction (Figure 11d). The crush load from the numerical simulation could efficiently predict the experimental results, despite the slight difference with time delay of the second peak between the numerical simulation and the experiment. These differences could be attributed to the following: First, the lattice structure was manually manufactured and assembled, which inevitably introduced geometry errors, including machining errors, assembly errors, size errors, defects, and so on. Euler buckling was known to be extremely sensitive to these geometry errors, which failed to easily perform quantitative evaluation and thus was not considered in the FEM models, leading to an error in crushing response prediction. Second, the damage initiation and evolution model was not considered in the FEM model. The development of an accurate, efficient, and robust damage model remained a challenge. Accordingly, the developed simplified Johnson–Cook model without damage initiation and evolution could not obtain a highly precise failure prediction under complex contact interfaces and stress state.

However, considering the complexity of the actual structure, the accuracy of numerical simulation could be acceptable. Thus, adequate confidence in the present numerical studies with the developed simplified Johnson–Cook model could be extended to engineering structure analysis.

## 5. Conclusions

Flax fiber-reinforced composites were expected to play an increasingly important role in the design of engineering structures subject to dynamic loadings because of the requirements for lightweight, low cost, recyclability, and excellent mechanical properties. Thus, full understanding of the dynamic material behavior of FFRCs became a priority. In this study, FFRCs were fabricated by VARI. The effects of strain rate on the mechanical properties of the FFRCs were investigated using quasi-static and SHPB experiments. Distinguishing strain-hardening behaviors were observed under both quasi-static and dynamic loading conditions, which revealed that FFRCs exhibited evident strain-rate sensitivity. On the basis of the experimental results, a simplified Johnson–Cook model was obtained and verified by numerical simulation of SHPB experiments. Moreover, the dynamic behavior of the lattice structures composed of FFRCs was numerically simulated and compared with the quasi-static and drop-hammer crushing experiments. The results evidently revealed that the proposed simplified Johnson–Cook model was able to accurately describe the dynamic mechanical behaviors of the FFRC material. Overall, the results of this study could be a solid step to elucidate the dynamic mechanical behaviors of the FFRC material and could provide valuable guidance for future applications in engineering.

## Figures and Tables

**Table 1:** Yield strength of representative curves.

$\dot{\varepsilon }$	0.006 s^−1^	0.06 s^−1^	0.6 s^−1^	1300 s^−1^	2200 s^−1^
$\sigma_{1}$	102.0 MPa	112.3 MPa	149.9 MPa	152.0 MPa	163.7 MPa

**Table 2:** Fitting parameters in the simplified Johnson–Cook model.

Simplified Johnson-Cook model	A (MPa)	B (MPa)	n	C	${\dot{\varepsilon }}_{0}$
Value	102.0	70.8	0.416	0.047	0.006

**Table 3:** Comparison of peak load between numerical simulation and experiments.

Impact Energy (J)	Peak Load (kN)					Deviation %
	Experiments				Simulation	
	No. 1	No. 2	No. 3	Mean		
Quasi static	10.89	11.18	11.27	11.11 ± 0.16	11.73	5.58
30 J	15.13	18.74	12.79	15.55 ± 2.45	17.96	15.50
45 J	16.70	14.06	-	15.83 ± 1.32	18.40	16.23

## Supplementary Materials

The following are available online at https://www.mdpi.com/1996-1944/12/6/854/s1, Figure S1: Tensile stress-strain curves of pure matrix.
