# Supplementary material for: Mechanical Behaviors of Flax Fiber-Reinforced Composites at Different Strain Rates and Rate-Dependent Constitutive Model
Source: Materials (Basel). 2019 Mar 13;12(6):854. doi: 10.3390/ma12060854 (PMC6471640; doi:10.3390/ma12060854)
Supplement: Supplementary file 1 [file materials-12-00854-s001.pdf]

materials

Supplementary Materials

Mechanical Behaviors of Flax Fiber-Reinforced Composites at Different Strain Rates and Rate-Dependent Constitutive Model

Dayong Hu [1,2], Linwei Dang [1,2], Chong Zhang [1,2] and Zhiqiang Zhang [3,4,*]

1 Department of Aircraft Airworthiness Engineering, School of Transportation Science and Engineering, Beihang University, Beijing 100191, China; hudayong@buaa.edu.cn (D.H.); danglinwei@buaa.edu.cn (L.D.); chongzhang@buaa.edu.cn (C.Z.)
2 Aircraft/Engine Integrated System Safety Beijing Key Laboratory, Beijing 100191, China
3 Beijing Key Laboratory of Rehabilitation Technical Aids for Old-Age Disability, Key Laboratory of Rehabilitation Technical Aids Analysis and Identification of the Ministry of Civil Affairs, National Research Center for Rehabilitation Technical Aids, Beijing 100176, China
4 Qinhuangdao Institute of National Research Center for Rehabilitation Technical Aids, Qinhuangdao 066000, China
* Correspondence: zhangzhiqiang@nrcrta.cn;

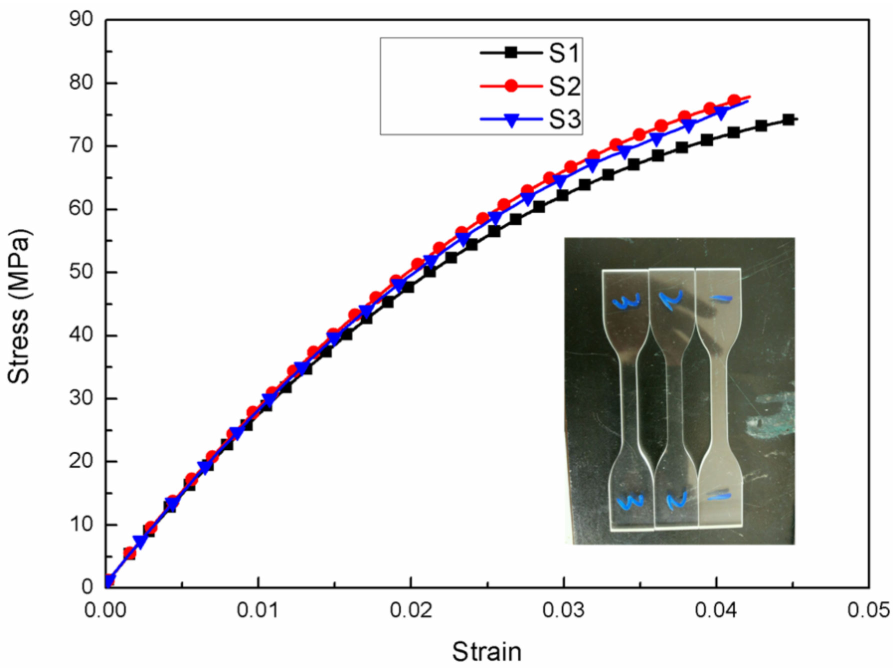

Figure S1. Tensile stress-strain curves of pure matrix.
